# Data on the levels of Melamine- migration from Melamine- ware products and effect of food type and time on it

**DOI:** 10.1016/j.dib.2018.10.039

**Published:** 2018-10-17

**Authors:** Ehsan Haghi, Mahmood Alimohammadi, Sahar Asadnejad, Fariba Razeghi, Parisa Sadighara

**Affiliations:** a Department of Environmental Health Engineering, Food Safety Division, School of Public Health and Center for Environmental Research, Tehran University of Medical Sciences, Tehran, Iran; b Department of Environmental Health Engineering, School of Public Health and Center for Environmental Research, Tehran University of Medical Sciences, Tehran, Iran

**Keywords:** Melamine, Migration, Spectrophotometer, Temperature

## Abstract

Nowadays, Melamine- containers is widely use to because of heat- resistant. Due to the effects of Melamine- on human health, constant and long-term usage of Melamine- containers can be a source of Melamine- exposure to human body. The objective of this research was to measure the levels of Melamine- migration from Melamine- ware-products into foods at different test conditions and Effect of food type and Time on it. Spectrophotometer UV/VIS method was used to detect the limits of Melamine- and the method was based on the in the complex of Melamine- formaldehyde and Uranin (a ketone group).The limit of detection (LOD) of the method was 0.2 (µg/ml) which is functional for measuring. Migration was less than the standard level of European Union (30 µg/ml). In this study, 3% acetic acid, distilled water and 15% ethanol were used as simulants. The results showed the temperature is an important factor in Melamine- migration and in 97% of cases, with increasing temperature from 30 to 90 there is a significant increase (*P* < 0.05) in Melamine- migration furthermore migration from acidic simulants was more than alcoholic and neutral ones (*p* < 0.001).

**Specifications table**

**Table:** 

Subject area	Food science
More specific subject area	Food migration
Type of data	Table, figure
How data was acquired	Melamine- migration were quantified using spectrophotometric method
Data format	analyzed
Experimental factors	– 3% acetic acid, distilled water and 15% ethanol were heated before exposing to samples
	– Melamine- ware-products were exposed to food simulants by filling with food simulants for 30 and 90 min
Experimental features	Melamine- migration measurement
Data source location	Tehran, Iran
Data accessibility	The data is within this article
Related research article	*Not available*

**Value of the data**

• The data presented in this article, describes the quantitative determination of Melamine- migration from Melamine- wares at different test conditions.
• The data in this article provides information about the effect of temperature and pH on the rate of Melamine- migration from Melamine- ware-products into foods.
• The given data shows the significant effect of high temperature on the Melamine- migration.

## 1 Data

**Fig. 1:**
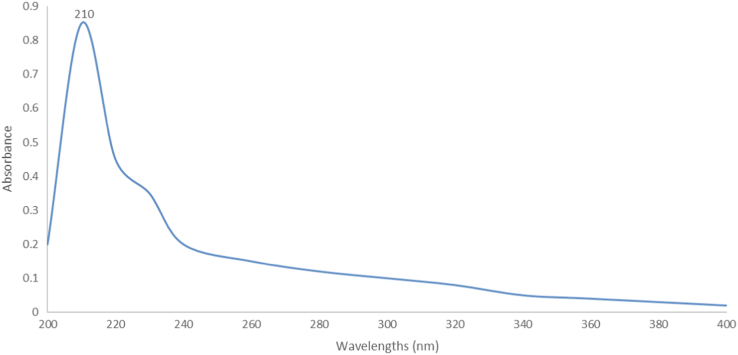
The scan of absorption spectrum of Melamine–Uranin- formaldehyde complex within the UV range.

**Fig. 2:**
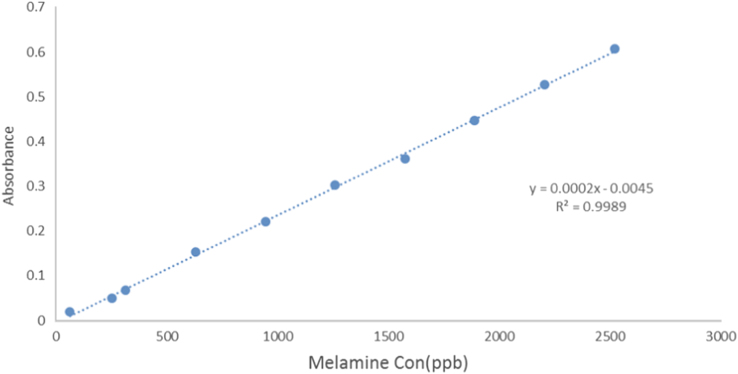
Calibration curve of Melamine– Uranine – formaldehyde complex.

This article presents data on the levels of Melamine- in test items exposed to food simulants under different test conditions (Table 1, Table 2). The absorption spectrum of Melamine–Uranin- formaldehyde complex within the UV range is given in Fig. 1. Calibration curve of the method was shown in Fig. 2.

**Table 1:** The results of measuring the migration rate of samples by spectrophotometric method at a temperature of 30°C.

**Sample**	**Test**		**Mean Result** ± **SD (ppm)**
**Sample 1**	Water 30°C - 90 min		0.635 ± 0.096
	Acetic acid (3%) 30°C - 90 min		2.061 ± 0.070
	Ethanol (15%) 30°C - 90 min		2.796 ± 0.052
**Sample 2**	Water 30°C - 90 min		2.333 ± 0.038
	Acetic acid (3%) 30°C - 90 min		1.214 ± 0.011
	Ethanol (15%) 30°C - 90 min		1.310 ± 0.023
**Sample 3**	Water 30°C - 90 min		1.717 ± 0.020
	Acetic acid (3%) 30°C - 90 min		4.371 ± 0.014
	Ethanol (15%) 30 °C - 90 min		2.248 ± 0.040
**Sample 4**	Water 30 °C - 90 min		3.677 ± 0.028
	Acetic acid (3%) 30°C - 90 min		4.134 ± 0.065
	Ethanol (15%) 30°C - 90 min		1.934 ± 0.006

**Table 2:** The results of measuring the migration of samples by spectrophotometric method at a temperature of 90°C.

**Sample**	**Test**	**Mean Result + SD (ppm)**
**Sample 1**	Water 90°C - 90 min	1.342 + 0.030
	Acetic acid (3%) 90°C - 90 min	3.242 + 0.046
	Ethanol (15%) 90°C - 90 min	2.830 + 0.016
**Sample 2**	Water 90°C - 90 min	2.855 + 0.037
	Acetic acid (3%) 90°C - 90 min	5.628 + 0.017
	Ethanol (15%) 90°C - 90 min	5.011 + 0.007
**Sample 3**	Water 90°C - 90 min	2.738 + 0.039
	Acetic acid (3%) 90°C - 90 min	6.750 + 0.054
	Ethanol (15%) 90°C - 90 min	4.965 + 0.055
**Sample 4**	Water 90 °C - 90 min	4.731 + 0.055
	Acetic acid (3%) 90°C - 90 min	6.281 + 0.036
	Ethanol (15%) 90°C - 90 min	2.441 + 0.021

## 2 Experimental design, materials, and methods

### 2.1 Materials

Melamine- (Sigma-Germany-Lot number: 1422105v)- Uranin (C20H10Na2O5) (Sigma-Germany)- formaldehyde (Merck-Germany)- deionized distilled water.

Spectrophotometry UV/VIS (America-Perkinelmer)- Scale with microgram detection limit (Sartorius-Germany)- Ultrasonic device (Elma-Germany)- Sampler (Eppendorf-Germany). 3% acetic acid, 15% ethanol and deionized distilled water were used as food simulants for quantifying melamine migration.

### 2.2 Making the solutions and plotting the calibration curve

Melamine- stock solution with a concentration of 6.3 μg/ml was prepared along with one solution of Uranin with a concentration of 6.3 μg/ml. This was followed by preparing pure formaldehyde which was another component of the intended complex.

To plot the calibration curve, 10 solutions were mixed with different volumes of stock Melamine- (0.05–2 ml) with 0.5 ml of Uranin solution as ketone and 1 ml of pure formaldehyde. Then, different volumes of deionized distilled water were added to each of the solutions to reach a final volume of 5 ml In the final solution, the concentration of Uranin and formaldehyde was fixed, while the concentration of Melamine- varied between 0.063 and 2.52 μg/ml. Then, absorption of the solutions was measured by the device and calibration curve was plotted (Fig. 2). Three replications were considered for each concentration.

### 2.3 Making and measuring the sample absorption

The sample filled up to 1 cm off the edge with the solutions of simulants, formerly reaching 90 °C. To keep the proposed temperature of the simulants during contact with the tableware, the samples were put in an oven with the desired temperature.

In the next step, to measure absorption of each sample, 3.5 ml of it was transferred to the test tube and 0.5 ml of Uranin plus 1 ml of formaldehyde were added to it. After combining, they were placed in the spectrophotometry device to measure their absorption.

To measure the absorption of the samples, the absorption of Melamine–Uranin- formaldehyde complex, formed from Mannich reaction, was first scanned within the range of UV (200–400 nm). After that, a wavelength, at which the complex had the maximum absorption, was specified (Fig. 1). The device became zero with a solution, containing all available materials in the sample except for Melamine-. The absorption of the samples was measured at the wavelength with the maximum absorption of M-F-U complex.

#### 2.3.1 Extraction procedure

The total of 24^1^ samples of Melamine- wares were exposed to 3% acetic acid, distilled water and 15% ethanol at different test conditions (30 °C, 90 °C) by filling with food simulants for 30 and 90 min (3 repetition for each sample). Food simulants were heated before exposing to samples. 3.5 ml of food simulant was injected into spectrophotometry device. The melamine migrations to simulants were calculated. The mean of three replicates for each sample was recorded in Tables 1 and 2. [1], [2], [3], [4], [5], [6].

## Footnotes

1 4: number of Melamine–wares. 3: condition tests. 2: different temperatures.
